# Patient outcomes and session dose in a randomized controlled trial of the Transdiagnostic Intervention for Sleep and Circadian Dysfunction: Influential factors and methodological considerations

**DOI:** 10.1016/j.brat.2026.104987

**Published:** 2026-02-17

**Authors:** Anne E. Milner, Laurel D. Sarfan, Marlen Diaz, Rafael Esteva Hache, Estephania Ovalle Patino, Allison G. Harvey

**Affiliations:** a University of California, Berkeley, CA, USA; b Department of Research and Evaluation, Kaiser Permanente Southern California, Pasadena, CA, USA; c Stony Brook University, New York, NY, USA; d University of California, Los Angeles, CA, USA

**Keywords:** Evidence-based psychological treatment, Transdiagnostic, Design considerations, Dose-response

## Abstract

**Objective:**

Randomized controlled trials (RCTs) assessing the implementation of evidence-based psychological treatments (EBPTs) into clinical practice may involve variations that are not always systematically investigated. This study aimed to investigate the impact of such variations in the context of an effectiveness-implementation hybrid trial - specifically, the relationship between trial outcomes and number of treatment sessions, dropout, and differences in assessment timing.

**Method:**

Adults (*N* = 539) with sleep and circadian problems were randomized to the Transdiagnostic Intervention for Sleep and Circadian Dysfunction (TSC; Adapted or Standard version) or usual care followed by delayed treatment (UC-DT).

**Results:**

Participants who completed the recommended number of TSC sessions showed larger effect sizes and more significant improvements relative to UC-DT, when compared to participants who received a partial session dose. Notably, a substantial number of participants who dropped out of treatment showed a meaningful clinical change by mid-treatment (20%), though this number did not differ significantly from the number of patients who received the full dose of treatment. Larger treatment effects were observed when assessments were completed within 3 months of their planned date. Kaplan-Meier Survival Analyses indicated that the dose to optimize the primary outcome of sleep disturbance at post-treatment was eight sessions for Standard TSC and six sessions for Adapted TSC.

**Conclusions:**

This study underscores the impact of methodological variations on treatment outcomes in the implementation of TSC into clinical practice. Furthermore, our findings establish specific session thresholds for Standard and Adapted TSC to optimize clinical outcomes.

## 1. Introduction

Evidence-based psychological treatments (EBPTs) are widely recommended as first-line treatments for a range of mental health problems (Clark, 2018; Division 12 of the American Psychological Association, 2016; National Institute for Health and Care Excellence, 2020). EBPTs are informed by rigorous scientific research and are designed to target psychological and behavioral factors that contribute to the cause and/or maintenance of mental health challenges (Barlow et al., 2013; Chambless & Hollon, 1998). Despite their proven effectiveness, there are several gaps in knowledge.

First, there is limited research exploring how variations in the implementation of EBPTs into clinical practice influence outcomes. For example, there is a positive association between the number of sessions a patient attends and improvements in treatment outcomes (Kadera et al., 1996; Strassberg et al., 1977). Indeed, intent-to-treat analyses include all participants who are randomized to treatment condition in analyses (Gupta, 2011), while per-protocol analyses are commonly conducted to assess data only from participants who complete the study and adhere to treatment protocols (Andrade, 2022). However, sub-group analyses that include only those participants who receive a partial dose are rarely reported. Consequently, it remains unclear whether participants who received a “dose” of treatment sessions that differ from the intended treatment protocol, including those who drop out, still experience any treatment benefit.

Second and related, past research has shown that many patients who drop out of treatment display significant improvements in outcomes (Szafranski et al., 2017). This finding suggests that even those who attend fewer sessions may experience significant improvements and potentially discontinue treatment due to symptom improvement. In other words, it is possible that a subset of participants do not require a full course of treatment. Identifying these participants represents an important step toward tailoring treatment dose to individual needs, which in turn could optimize resource allocation, reduce patient burden, and ultimately improve the efficiency and accessibility of care.

Third, in trials testing the implementation of EBPTs in routine clinical practice settings, flexibility may be needed to balance the needs of non-academic partners and participants with study rigor (Kendall & Frank, 2018; Wiltsey Stirman et al., 2017). One area where this may be important is assessment timing. For example, ‘real world’ participants receiving treatment in community settings may have more barriers to research participation (Levinson Miller et al., 2003). As a result, it may be more difficult for them to complete assessments along the strictly monitored timeframes in a typical randomized controlled trial (RCT). That said, variations in the timing of follow-up assessments in RCTs assessing the implementation of EBPTs could lead to inconsistencies in observed intervention effects. To our knowledge, no previous research has explicitly investigated how the timing of planned follow-up assessments impacts clinical trial outcomes. However, given that intervention effects typically diminish over time (e.g., Johnsen & Friborg, 2015), the timing of assessments may indeed play a role in the observed outcomes.

Fourth, there is a need to determine the optimal number of treatment sessions needed to maximize treatment outcomes. The National Institute of Mental Health has emphasized the importance of determining the optimal dose of psychosocial treatments, and that this process should be approached with the same level of rigor as determining the dose in psychopharmacological interventions (Insel & Gogtay, 2014).

The present study is a secondary analysis of a randomized controlled trial (referred to henceforth as the “parent trial” (Harvey et al., 2025, 2026), in which the EBPT delivered was the Transdiagnostic Intervention for Sleep and Circadian Dysfunction (TSC; Harvey & Buysse, 2017). TSC targets a range of sleep and circadian problems experienced by people with a range of serious mental illness (SMI) diagnoses and has been previously associated with improvements in sleep outcomes relative to usual care (Harvey et al., 2021). Previous research examining the optimal number of sessions for Cognitive Behavior Therapy for Insomnia (CBT-I) found that four sessions optimized outcomes (Edinger et al., 2007). However, the dose of TSC needed to optimize clinically meaningful improvements remains unclear. Indeed, it is possible that the ideal dose is different to CBT-I, given that TSC targets a broad range of sleep and circadian problems experienced by people diagnosed with a range of SMI.

The parent trial tested two versions of TSC: Standard TSC and an adapted version, referred to as Adapted TSC, designed to improve “fit” between TSC and its implementation into community mental health centers (CMHCs). Adapted TSC was customized to fit the CMHC context by incorporating theory, previous data, and input from end-users of TSC (Armstrong, Aguilera, et al., 2022; Gumport et al., 2019, 2020; Harvey et al., 2021; Kilbourne et al., 2007). In the parent trial, participants were randomized to receive TSC immediately or usual care followed by TSC after a 4-week delay for Adapted and an 8-week delay for Standard TSC (UC-DT). The parent trial also evaluated the Train-the-Trainer (TTT) approach, a method for implementing and scaling training, where expert university-based providers trained an initial cohort of CMHC providers (Generation 1), who then became local trainers and trained the next generation of providers (Generation 2). Relative to UC-DT, TSC was associated with improvements across the main primary and secondary outcomes, and there were no differences in outcomes between Adapted and Standard TSC for both Generation 1 (Harvey et al., 2025) and Generation 2 (Harvey et al., 2026). However, there were signals that Adapted may have advantages, at least in routine practice settings like CMHCs, as it is a briefer version of TSC that still results in significant improvements in outcomes. Additionally, there was lower provider recruitment and higher patient drop-out in Standard relative to Adapted TSC (Harvey et al., 2025).

The present study is a secondary analysis exploring the potential impact of several factors on the findings of the parent trial. These factors include three potential variations in the parent trial that assess the implementation of TSC into routine practice, which were further influenced by the COVID-19 pandemic and the CMHC context in the parent trial. The specific factors investigated in the current study were the number of sessions attended, the response to treatment of participants who dropped out, and differences in the timing of assessments (Aims 1–3). We also sought to determine the optimal number of sessions required to observe a meaningful clinical change in the outcome (Aim 4).

The first aim was to evaluate whether the number of treatment sessions completed influenced outcomes. In the present study, sessions were delivered on a weekly schedule; thus, the current analyses focus on number of sessions completed, rather than session frequency. We tested the effectiveness of TSC, compared to UC-DT – mirroring the parent trial analyses – by the number of sessions attended to evaluate whether outcomes differed by number of sessions completed (Aim 1a). Aim 1b tested whether, among these different sub-groups, TSC treatment condition (Adapted versus Standard TSC) was associated with fit to the CMHC context, operationalized as provider ratings of acceptability, feasibility, and appropriateness. The second aim was to evaluate whether participants who dropped out of treatment demonstrated clinically meaningful improvements at mid-treatment or post-treatment assessments, and whether meaningful improvement differed between those who remained in treatment versus those who dropped out. Aim 3 was to explore whether variability in the timing of assessment dates across participants influenced the results. To address this, the analyses specified in Aim 1a were repeated, stratifying participants by completion of the post-treatment assessment either within or after 3 months of the target assessment date. Aim 4 was to establish the number of sessions of Standard and Adapted TSC needed to optimize meaningful change in outcomes at post-treatment and six-month follow-up (6FU). The latter is important as providers were initially trained to deliver 4 sessions of Adapted TSC and 8 sessions of Standard TSC; in practice, providers delivered an average of 4.99 sessions for Adapted TSC (SD = 1.91) and 8.95 sessions for Standard TSC (SD = 8.01). This deviation in the number of sessions delivered raises the possibility that the provider may have perceived a need for a greater number of sessions. Such variability offers a valuable opportunity to examine the optimal number of sessions required within each TSC condition to achieve clinically meaningful outcomes.

## 2. Method

Participants included in this study were drawn from an RCT funded by the National Institute of Mental Health (R01MH120147). The current study focuses on Generation 1 (Harvey et al., 2025) and 2 (Harvey et al., 2026), the Implementation Phase and the Train-the-Trainer Phase. Both phases tested the effectiveness outcomes of Standard and Adapted TSC. To maximize the sample size and because findings were similar across both generations, we collapsed over these two phases. See Sarfan et al. (2023) and Callaway et al. (2023) for full protocol details on Phase 1 and Phase 2 of the parent trial, respectively.

### 2.1. Participants

CMHC providers and participants were recruited from the following ten counties in California, USA: Alameda, Contra Costa, King, Monterey, Placer, Santa Cruz, Solano, Santa Clara, Santa Barbara, and Lake. The participants were 177 CMHC providers (Standard = 53, Adapted = 124), and 539 CMHC patients who met the criteria for sleep and circadian dysfunction. Of the patients, 276 were randomized to receive TSC immediately, and 263 were randomized to UC-DT. The parent trial was approved by the Committee for the Protection of Human Subjects at the University of California, Berkeley. Providers and patients provided written informed consent before participating in the study. See Table 1 for participant demographics by treatment condition (Adapted vs. Standard TSC). See Supplement Table 1 for participant demographics by TSC condition (UC-DT vs. immediate TSC) and Supplement Table 2 for provider demographics. Inclusion and exclusion criteria for the parent RCT are reported in Supplement File 1.

### 2.2. Treatments

Two variations of TSC were tested: Standard TSC and Adapted TSC. The control condition was usual care followed by delayed treatment with Adapted or Standard TSC (UC-DT). Sites were cluster-randomized by county to Adapted or Standard TSC with 1:1 allocation. Then, within each county, patients were randomized to immediate TSC or UC-DT. Supplement Table 3 summarizes the treatment modules for the Standard and Adapted conditions.

#### 2.2.1. Standard TSC

Standard TSC was delivered by CMHC providers across eight 50-min, weekly sessions (Harvey & Buysse, 2017). It was comprised of 4 cross-cutting modules featured in every session, 4 core modules, and 7 optional modules based on clinical presentation, treatment goals, and provider case conceptualization. Training for the Standard TSC condition consisted of a 1-day workshop (i.e., 6–8 h) or two, 3-h training blocks, based on CMHC preference.

#### 2.2.2. Adapted TSC

Adapted TSC was delivered by CMHC staff across four, 20-min, weekly sessions. Treatment consisted of the same four *cross-cutting modules* as in Standard TSC as well as three of the core modules and one of the optional modules. Training for the Adapted TSC condition consisted of four, 1-h workshops or two, 2-h workshops, based on CMHC preferences.

#### 2.2.3. UC-DT

In UC-DT, patients began with usual care for four or eight weeks, depending on whether their CMHC was randomized to Adapted TSC or Standard TSC, respectively. After the delay, they received Adapted or Standard TSC, based on the condition to which their CMHC had been randomized. In the CMHCs, usual care consists of working with a service provider (e.g., psychologist, case manager, occupational therapist, psychiatrist, nurse practitioner) who provided direct mental health support alongside other services as needed (e.g., housing support).

### 2.3. Measures

The data were collected as part of the NIMH parent trial (R01MH120147). Only measures that were included in the present secondary analysis are reported below. In addition to the measures below, a sociodemographic form was completed by providers and patients.

#### 2.3.1. Providers

##### Acceptability, Appropriateness, and Feasibility.

Providers rated the acceptability, appropriateness, and feasibility of TSC via the *Acceptability of Intervention Measure* (AIM), *Feasibility of Intervention Measure* (FIM), and *Intervention Appropriateness Measure* (IAM) (Weiner et al., 2017). This 4-item measure was rated on a scale from 1 (completely disagree) to 5 (completely agree). This measure has demonstrated satisfactory validity, internal reliability, test-retest reliability, and sensitivity to change (Weiner et al., 2017).

##### Session Dose.

Providers logged the number and length of each session delivered each week through a HIPAA-compliant online survey. We examined two separate variables (1) *Number of Sessions* and (2) *Cumulative Length of Sessions*. For the number of sessions, 507 participants had available data, and 7 outliers were removed, resulting in a final sample of 500. For cumulative session length, any provider who had missing session length data for any session within a participant was not included in the analyses, resulting in a final sample of 326.

#### 2.3.2. Patients

##### Sleep Disturbance.

The 8-item PROMIS-Sleep Disturbance (PROMIS-SD) was used to assess disruption to sleep (e.g., trouble staying asleep) over the past seven days (Yu et al., 2012). Items were rated on a scale from 1 (not at all/never/very poor) to 5 (very much/always/very good). T-scores were calculated by summing the raw scores and using conversion tables from healthmeasures.net. To aid interpretability and comparability across studies and PROMIS measures, scores are converted to a standardized (*M* = 50, *SD* = 10. A t-score of 50 represents the mean of the United States general population, based on calibration testing with a large reference sample. Converted t-scores range from 28.9 to 76.5, with higher scores indicating greater sleep disturbance. This measure has demonstrated acceptable reliability and validity (Buysse et al., 2010; Yu et al., 2012).

##### Sleep-Related Impairment.

The 8-item PROMIS-Sleep Related Impairment (PROMIS-SRI) was used to assess perceived sleep problems during waking hours (e.g., alertness, sleepiness, tiredness) over the past seven days and was assessed using the same scale and scoring as the PROMIS-SD. Converted t-scores for the PROMIS-SRI range from 30.0 to 80.1, with higher scores indicating greater sleep-related impairment. This measure has demonstrated adequate reliability and validity (Yu et al., 2012).

##### Overall Sleep Health.

The Sleep Health Composite captures overall sleep health for the complexity of sleep problems in SMI that are covered by TSC (SHC; Dong et al., 2019). It is defined as the sum of scores on six sleep health dimensions: Regularity (midpoint fluctuation), Timing (mean midpoint), Efficiency (sleep efficiency), Duration (total sleep time), Satisfaction (sleep quality question on PROMIS-SD), and Alertness (daytime sleepiness question on PROMIS-SRI). All dimensions – except Satisfaction and Alertness – were assessed via questions about sleep-wake patterns over the past seven days. The satisfaction and alertness dimensions were drawn from questions on the PROMIS-SD and PROMIS-SRI scales, respectively. Each dimension is dichotomized as either 0 = poor or 1 = good, based on established literature and/or recommendations/consensus (Dong et al., 2019). Total sum scores range from 0 to 6, where higher scores indicate better sleep health. Initial validity of this measure has been established (Dong et al., 2019).

##### Functional Impairment.

Functional impairment was assessed via the Sheehan Disability Scale (SDS; Sheehan et al., 1996). Impairment in work and school, social life, and home and family were rated via three items on a scale from 0 (not at all) to 10 (extremely). Items are summed to produce a single score. Scores ranged from 0 to 30, with higher scores indicating greater functional impairment. This measure demonstrated good reliability and validity (Leon et al., 1997; Sheehan et al., 1996).

##### Psychiatric Symptoms.

The DSM-5 Cross-Cutting Measure assessed psychiatric symptoms across 13 mental health domains (Narrow et al., 2013). Participants rated how often they were bothered by each symptom on a scale from 0 (not at all) to 4 (nearly every day). Scores ranged from 0 to 52, with higher scores indicating more severe symptoms. This measure has demonstrated good test-retest reliability and clinical utility (Clarke & Kuhl, 2014; Narrow et al., 2013).

### 2.4. Procedure

CHMCs and patients were randomized through a computerized randomization sequence. When randomizing patients, we stratified for presence of psychosis or not (current), presence of substance use or not (current), and age (≥50 or not), as there was evidence that these variables can impact sleep or treatment outcome (Armstrong, Dong, & Harvey, 2022; Harrow et al., 2000; Large et al., 2014). The data collection coincided with the COVID-19 pandemic, so most study processes were conducted virtually.

Providers and patients were consented by the assessment team prior to participation. All patients were compensated for their participation, and providers were compensated if permitted by their CMHC. The assessments were completed by the assessment team, comprised of experienced assessors. Patient assessments for the immediate TSC condition were completed at pre-treatment, post-treatment, and six months after treatment (6FU). A mid-treatment assessment was also conducted that measured select outcome variables (PROMIS-SD, PROMIS-SRI, and SHC). Patient assessments in the UC-DT condition were completed at pre-treatment and four or eight weeks after pre-treatment (i.e., at the end of usual care and before delayed treatment with TSC, referred to as “post UC-DT”), depending on whether their county had been randomized to Adapted or Standard TSC, respectively. Patients did not complete a mid-assessment or 6FU assessment after the delay portion of the UC-DT. The latter was a compromise made with CMHC partners so that patients would have minimal wait time before receiving treatment. As a result, patients started delayed treatment with TSC after the post UC-DT assessment. In delayed TSC treatment, patients completed the same assessments as those in the immediate TSC condition i.e., pre-treatment, post-treatment, mid-treatment, and 6FU. Provider assessments were completed one time after the providers completed TSC training (i.e., post-training), as well as at mid-treatment and post-treatment for each patient they treated.

### 2.5. Analysis plan

All data analyses were conducted in R (R Core Team, 2021). Aim 1a and 3 analyses compared Immediate TSC to UC-DT. The patient assessments for immediate TSC at pre-treatment and post-treatment were used. For UC-DT, the patient assessments for pre-treatment and post UC-DT (i.e., before delayed treatment) were used. Analyses not comparing UC-DT to immediate TSC (i.e., Aims 1b, 2, and 4) combined data from the delayed treatment phase of UC-DT with the immediate TSC condition, such that TSC data from both groups were analyzed. Specifically, pre-treatment data from both groups, along with post-treatment data from the UC-DT group following TSC treatment and equivalent timepoints from the Immediate TSC group, were included in the analysis. The rationale for this decision is threefold 1) the treatment contents were the same for the two conditions, 2) the only difference between the two conditions was that those in the UC-DT group received usual care until starting TSC after a delay period, and 3) to increase sample size and achieve greater statistical power.

**Multilevel Models (Aims 1a, 1b, and 3).** For Aims 1a, 1b, & 3, multilevel models (MLMs) were used to account for multiple observations nested within a patient or provider. All MLMs used maximum likelihood estimation (MLE) to handle missing data under the missing at random assumption and robust standard errors. Effect sizes for all multilevel models are represented with ‘*d*’ and were calculated using unadjusted change scores and raw standard deviations at pre-treatment from each treatment condition (Feingold, 2009). All MLMs compared pre-treatment/training to post-treatment and, for level 1, included a dummy-coded time indicator as the predictor (1 = post-treatment, pre-treatment/training as the reference). For all MLMs, the level 2 equation included dummy-coded treatment condition (*Aim 1a, 3:* 1 = immediate TSC, with UC-DT as the reference; *Aim 1b:* 1 = Adapted TSC, with Standard as reference) and treatment-by-time interaction terms, which were the parameters of interest. For Aims 1a and 3 MLMs, the outcomes were PROMIS-SD, PROMIS-SRI, Sleep Health Composite, DSM-5 Cross-Cutting, and SDS. For Aim 1b MLMs, the outcomes were provider ratings of acceptability (AIM), Appropriateness (IAM), and feasibility (FIM).

**Chi-Square (Aim 2).** Chi-squared tests were used to assess whether the proportion of participants who demonstrated a clinically meaningful change at mid-treatment and post-treatment differed between those who dropped out of treatment and those who completed treatment. Drop-out was operationalized as a binary variable based on treatment dosage (1) those who completed at least four sessions of Adapted TSC or eight sessions of Standard TSC were classified as receiving a full dosage, and (2) those who completed at least one session but fewer than the required number were classified as having received a partial dosage (i.e., dropout). Clinically meaningful change was determined for each participant using a reliable change index (RCI) for each outcome, calculated from pre-to mid-treatment and post-treatment (Jacobson & Truax, 1992). Participants were classified as having achieved clinically meaningful change if their RCI exceeded the critical value of ±1.96. This resulted in a dichotomous variable where 0 = no clinically meaningful change, and 1 = clinically meaningful change.

**Kaplan-Meier Survival Analyses (Aim 4).** Kaplan-Meier Survival Analyses were conducted to estimate the number of treatment sessions associated with clinically meaningful improvement in patient outcomes at post-treatment and 6FU. The event of interest was defined as achieving a clinically meaningful improvement in sleep disturbance, sleep-related impairment, sleep health, psychiatric symptoms, or functional impairment based on the RCI method described above (0 = no clinically meaningful change, and 1 = clinically meaningful change). Time-to-event was operationalized as the number of treatment sessions completed prior to achieving this improvement, examined as a function of TSC condition (Standard vs. Adapted). There were seven outliers for the number of sessions participants received that were removed from the analyses. We report the median number of sessions associated with a 50% probability of achieving clinically meaningful improvement, as well as the number of sessions needed for 25% and 75% of participants to achieve clinically meaningful change. To compare the likelihood of improvement across treatment conditions, Cox regression models were used to estimate hazard ratios for not achieving a clinically meaningful change between the Adapted and Standard TSC conditions. For consistency with Horwitz et al. (*in preparation*), we also conducted the above analyses to estimate the optimal cumulative session length to observe clinically meaningful change in the outcome (see Supplement File 1).

#### 2.5.1. Covariates

Across MLM models, covariates included patient stratification variables (age, substance use, psychosis), county, and provider generation. Stratification variables were included as covariates to account for variables used in randomization, consistent with recommendations for the analysis of randomized trials (Kahan & Morris, 2012). County was entered as a fixed factor (dummy variable) rather than as a random effect due to the relatively small number of clusters, to account for differences between counties, which served as the unit of randomization to treatment condition. Provider generation was included as a covariate to account for differences in training, i.e., whether the provider was trained by UCB staff (Generation 1) or trained by a local trainer (Generation 2).

### 2.6. Transparency and openness

All research materials, data, and analysis code are available from the authors upon request. This secondary analysis was not pre-registered but uses data from a randomized controlled clinical trial that were pre-registered on clinicaltrials.gov (generation 1: https://clinicaltrials.gov/study/NCT04154631; generation 2: https://clinicaltrials.gov/study/NCT05805657). Raw data for outcomes have been uploaded onto the National Data Archive.

### 2.7. Power

This study was a secondary analysis that was not powered a priori; power calculations were determined for a previously reported RCT, which was designed to address different primary aims (see Callaway et al., 2023; Sarfan et al., 2023). However, the protocol did pre-specify exploratory analyses that are reported in Aims 1 and 3. Given these limitations, we emphasize effect sizes and confidence intervals rather than statistical significance alone.

## 3. Results

### 3.1. Aim 1: analyses by treatment completion sub-groups

Analyses for Aim 1 were conducted separately for the following groups: (1) the overall sample, (2) treatment completers, (3) patients who completed more than half of the number of suggested sessions (i.e., >2 for Adapted and >4 for Standard), and (4) patients who completed half or fewer of the suggested sessions.

### 3.2. Aim 1a MLM: comparing TSC versus UC-DT on patient outcomes for sub-groups

See Supplement Table 4 for means, standard deviations, and effect sizes by session completion sub-group and Table 2 for multilevel modeling results. For the overall sample and participants who completed all sessions, the effect sizes for pre-to post-treatment differences between treatment condition (TSC vs. UC-DT), on improvements in sleep disturbance, sleep-related impairment, sleep health composite, psychiatric symptoms, and overall functional impairment were medium to large (*d* = 0.52 to 1.32), and confidence intervals for all estimates excluded zero. Similarly, for participants who completed more than half of the suggested sessions, effect sizes remained medium to large across outcomes (*d* = 0.58 to 1.17), confidence intervals for all estimates excluded zero with the exception of psychiatric symptoms. For participants who completed half or less of the recommended sessions, effect sizes were in the small to large range across outcomes (*d* = 0.33 to 1.04), and confidence intervals included zero for all outcomes with the exception of sleep health composite and psychiatric symptoms, indicating substantial uncertainty in these effects. Compared to subgroups that completed more treatment sessions, effect sizes were generally smaller, suggesting attenuated improvements among participants who received a lower treatment dose.

### 3.3. Aim 1b: Standard versus adapted TSC on provider perceptions of fit

See Table 3 and Supplement Table 5. For the overall sample and participants who completed all sessions, effect sizes for differences between TSC condition (Standard versus Adapted) from pre to post-treatment on providers’ perceptions of acceptability, feasibility, or appropriateness were negligible to small (*d* = 0.03 to 0.25), with confidence intervals including zero for all outcomes. For participants who completed more than half the recommended sessions, effect sizes for the differences between TSC condition (Standard versus Adapted) on provider perceptions of acceptability, feasibility, and appropriateness were medium to large (*d* = 0.49 to 0.92), such that Adapted TSC was associated with decreases in provider ratings of acceptability, feasibility, and appropriateness from pre-to post-treatment. All confidence intervals included zero, except for acceptability. Similarly, for participants who completed half or fewer sessions of TSC, effect sizes for differences between Adapted TSC compared to Standard TSC on providers’ perceptions of acceptability, feasibility, and appropriateness were medium to large (*d* = 0.50 to 0.91); however, all confidence intervals included zero. As effect sizes were notably larger for participants who did not complete a full session dosage (*d* = −0.92 to −0.49) compared to treatment completers (*d* = −0.15 to 0.04), this effect suggests that for those who did not complete a full session dose, ratings of fit decreased from pre-to post-treatment for the Adapted relative to the Standard TSC condition.

### 3.4. Aim 2: Clinical improvement by drop-out

Some participants who dropped out of the study still completed the mid- and/or post-treatment assessment. Among the 266 participants who had mid-treatment data and completed at least one treatment session, 134 (50.4%) achieved a clinically meaningful change by mid-treatment, while 132 (49.6%) did not. Of the participants who were considered dropouts at mid-treatment, 20.15% showed a meaningful change in outcome relative to 29.85% for treatment completers. A chi-squared test revealed no significant association between mid-treatment and drop-out status (*χ*2(1) < 0.01, *p* = 0.95, OR = 1.07, 95% CI [0.61, 1.89]). Similarly, of 275 participants with post-treatment data who had completed at least one session, 165 (60%) achieved a clinically meaningful change, while 110 (40%) did not. No association between meaningful change at post-treatment and drop-out was found (*χ*2(1) < 0.01, *p* = 1.00), OR = 1.02, 95% CI [0.55, 1.90]). These findings suggest that achieving clinically meaningful improvement by mid- or post-treatment was not related to treatment drop-out.

### 3.5. Aim 3: Follow-up assessment timing on patient outcomes

For this aim, analyses were conducted with two subgroups of participants who completed the post-treatment assessment: (1) within 3 months of the target assessment date, and (2) more than 3 months after the target completion date. The target date of the post-treatment assessment was the day of the final TSC session. Of the 142 participants who completed a post-assessment in the Immediate TSC condition, 82 completed within 3 months of the target date (*M* = 46 days, range = 4 days before to 89 days from target), and 60 did not (*M* = 176 days, range = 91 to 636 days). See Supplement Table 6 for means, standard deviations, and effect sizes by subgroup.

See Table 4 for MLM results. In the subgroup that completed the post-treatment assessment within 3 months of the target date, effect sizes for the differences between treatment condition (TSC, relative to UC-DT), from pre-to post-treatment on improvements in sleep disturbance, sleep-related impairment, sleep health composite, psychiatric symptoms, and overall functional impairment were medium to large (*d* = 0.56 to 1.51) and all confidence intervals excluded zero. Similarly, in the subgroup that completed the post-assessment more than 3 months after the target date, effect sizes for the difference between treatment condition across outcomes were small to large (*d* = 0.25 to 1.12), all confidence intervals excluded zero except for the sleep health composite. Across outcomes, effect sizes were generally larger for participants who completed the post-treatment assessment within 3 months of the target date, except for sleep-related impairment, which showed comparable effect sizes across both subgroups.

### 3.6. Aim 4: Optimal session dosage

The results for cumulative session length are reported in Supplemental File 1 and Supplement Table 7. The results for the number of sessions, including Kaplan-Meier Survival Analyses, corresponding sensitivity analyses, and hazard ratios from Cox regression models, are presented in Table 5. For clinically meaningful improvement in sleep disturbance at post-treatment, the median effective number of sessions was 8 (95% CI [6, 9]) for Standard TSC, and 6 (95% CI [6, 7]) for Adapted TSC. For clinically meaningful improvement in sleep disturbance at 6-month follow-up, the median effective number of sessions was 7 (95% CI [6, 8]) for Standard TSC, and 6 (95% CI [5, 6]) for Adapted TSC. Cox regression analyses indicated that, relative to the Adapted TSC condition, participants in the Standard TSC condition were 44% more likely *not* to achieve a reliable, meaningful improvement in sleep disturbance at 6-month follow-up (Hazard Ratio = 1.38, *p* = 0.02). No significant effect of treatment condition was found at post-treatment.

For clinically meaningful improvement in sleep-related impairment at post-treatment, the median effective number of sessions was 8 (95% CI [7, NA]) for Standard TSC, and 7 (95% CI [6, 8]) for Adapted TSC. For clinically meaningful improvement in sleep-related impairment at 6-month follow-up, the median effective number of sessions was 8 (95% CI [7, 15]) for Standard TSC, and 6 (95% CI [6, 7]) for Adapted TSC. Cox regression analyses indicated that, relative to the Adapted TSC, participants in the Standard TSC were 79% more likely not to achieve a reliable, meaningful improvement in sleep disturbance at 6-month follow-up (Hazard Ratio = 1.78, *p* = 0.004). No significant effect of treatment condition was found at post-treatment.

For clinically meaningful improvement in the sleep health composite at post-treatment, the median effective number of sessions was 8 (95% CI [6, NA]) for Standard TSC, and 7 (95% CI [6, 8]) for Adapted TSC. For clinically meaningful improvement in sleep health composite at 6-month follow-up, the median effective number of sessions was 8 (95% CI [6, 10]) for Standard TSC, and 7 (95% CI [6, 8]) for Adapted TSC. No significant effects of treatment condition were found at post-treatment or 6FU.

For clinically meaningful improvement in psychiatric symptoms at post-treatment, the median effective number of sessions was 13 (95% CI [10, NA]) for Standard TSC, and 9 (95% CI [8, NA]) for Adapted TSC. For clinically meaningful improvement in psychiatric symptoms at 6-month follow-up, the median effective number of sessions was 11 (95% CI [8, NA]) for Standard TSC, and 9 (95% CI [7, NA]) for Adapted TSC. No significant effects of treatment condition were found at post-treatment or 6FU.

For clinically meaningful improvement in functional impairment at post-treatment, the median effective number of sessions was 11 (95% CI [8, NA]) for Standard TSC, and 7 (95% CI [7, 9]) for Adapted TSC. For clinically meaningful improvement in functional impairment at 6-month follow-up, the median effective number of sessions was 9 (95% CI [8, NA]) for Standard TSC, and 7 (95% CI [6, 8]) for Adapted TSC. Cox regression analyses indicated that, relative to the Adapted TSC condition, participants in the Standard TSC condition were 120% more likely to not achieve a reliable meaningful improvement in functional impairment at post-treatment (Hazard Ratio = 1.94, *p* = 0.01) and 78% more likely to not achieve a reliable improvement at 6-month follow-up (Hazard Ratio = 1.64, *p* = 0.01).

## 4. Discussion

The goal was to evaluate the potential impact of variations in an RCT assessing the implementation of TSC into routine clinical practice. We specifically investigated how factors such as the number of sessions completed, treatment response of participants who dropped out, and differences in assessment timing influenced the results of the parent trial. The variability in the number of sessions completed also allowed us to identify the optimal session dosage for achieving meaningful clinical changes in patient outcomes.

The first aim was to evaluate whether the number of treatment sessions completed influenced patient outcomes. Relative to UC-DT, TSC was generally effective across all session completion subgroups and outcomes. The exception was that for those who completed at least half of the recommended number of sessions, there were generally smaller effect sizes across outcomes (although all effect sizes were in the anticipated direction). Together, consistent with prior research (Harvey et al., 2021), these results suggest that TSC is an effective intervention for improving sleep, psychiatric, and functional impairment outcomes, particularly for those who complete a full dose of treatment. The finding that effect sizes were generally larger for full session dose raises the importance of adherence to the recommended number of sessions for maximizing the benefit of TSC. However, given that all effect sizes were at least small/medium—and ranging to large—in the anticipated direction, findings suggest that even an incomplete dosage of sleep and circadian treatment like TSC may have a positive impact on symptoms. However, the small effect for improvements in psychiatric symptoms for those who completed a partial session dose suggests that more sustained engagement of the primary target of sleep and circadian problems may be necessary to observe downstream effects, or that more explicit emphasis on strategies for psychiatric symptoms may be needed to shift this outcome.

Aim 1b tested whether TSC treatment condition (Adapted versus Standard TSC) was associated with fit to the CMHC context, operationalized as provider ratings of acceptability, feasibility, and appropriateness. For treatment completers, there were negligible to small effects of TSC condition (Adapted versus Standard) on provider perceptions of acceptability, feasibility, and appropriateness. However, for those who completed less than the full session dose, there were medium to large effects for the difference between Adapted and Standard TSC on provider ratings of acceptability, feasibility, and appropriateness, such that Adapted TSC was associated with decreases in provider ratings of acceptability, feasibility, and appropriateness from pre-to post-treatment. This suggests that when participants dropped out of treatment, Adapted TSC was rated by providers less favorably for treatment fit than Standard TSC. However, it’s important to note that dropout was higher in Standard TSC compared to the Adapted TSC condition. Perhaps the lower ratings of fit in the Adapted condition may reflect providers questioning the intervention’s fit more when patients dropped out of the briefer (4-session) Adapted TSC, compared to the longer (8-session) Standard TSC, where dropout was already more common. This aligns with research showing a discrepancy where clients expect to attend fewer treatment sessions than therapists (Mueller & Pekarik, 2000). Consequently, a dropout from the brief Adapted TSC might violate a provider’s implicit expectation of higher completion for an efficient protocol, thereby leading them to question its “fit” more critically.

For Aim 2, clinically meaningful improvement by mid- or post-treatment was found *not* to be significantly related to treatment dropout. However, a substantial number of participants who dropped out did show meaningful improvement by mid-treatment (20.15%), and this number did not significantly differ from those who completed the full course of treatment, consistent with prior research demonstrating that some participants who drop out of treatment show significant improvements in outcome (Szafranski et al., 2017). This has important implications for dropout interpretations and suggests that a better understanding of *why* participants drop out may be needed before reaching a conclusion (e.g., perhaps more dropouts do not necessarily mean that a treatment is less acceptable, but rather, that participants have experienced symptom improvement).

Aim 3 was to explore whether variability in the timing of the post-treatment assessment date across participants influenced the results. To our knowledge, the impact of the variation in the timing of assessments has not been directly assessed in prior research. Across those who completed the post-treatment assessment within 3 months and more than 3 months of their target date, there were medium to large effects for the difference between treatment condition (TSC vs. UC-DT) on all patient outcomes, with the exception of a small effect on sleep health for patients whose assessment was greater than 3 months after the target date. Effect sizes were larger for participants assessed within 3 months of the target post-treatment date in comparison to those who completed the assessment after 3 months. There are at least two interpretations of this finding. First, these results suggest that delays in the post-treatment assessment might diminish observed treatment effects. This underscores an important methodological consideration for future studies assessing the implementation of EBPTs into routine clinical practice to minimize assessment delays, ensuring a more robust evaluation of treatment efficacy, and enabling concrete conclusions across participants. Second, a self-selection bias may play a role, where individuals with less improvement may be more prone to delaying assessments. An important area for future research is to investigate the reasons for delays in the post-treatment and follow-up assessments, which might include ongoing symptom severity. Nevertheless, although there were larger effect sizes for the group that completed assessments within 3 months of the target date, there were still significant improvements for participants who had delayed assessments, suggesting that there is some flexibility in the timing of assessments. However, this may depend on the strength of the intervention relative to the comparison group.

Aim 4 was to establish the number of sessions of Standard and Adapted TSC needed to optimize meaningful change in the primary outcome of sleep disturbance, as well as secondary outcomes, at post-treatment and 6FU. At post-treatment, the median number of sessions required to achieve clinically meaningful improvement across sleep outcomes was 8 for Standard TSC and ranged from 6 to 7 for Adapted TSC. At 6FU, the median dose to achieve meaningful clinical change across sleep outcomes ranged from 7 to 8 sessions for Standard TSC and 6 to 7 sessions for Adapted TSC. Notably, at 6FU, Adapted TSC required significantly fewer sessions to achieve a clinically meaningful improvement in sleep disturbance and sleep-related impairment compared to Standard TSC. The median number of sessions required to achieve clinically meaningful improvement in psychiatric symptoms and functional impairment at post-treatment ranged from 11 to 13 for Standard TSC and 7 to 9 for Adapted TSC. For psychiatric symptoms and functional impairment at 6FU, the median was 11 sessions for Standard TSC and ranged from 7 to 9 sessions for Adapted TSC. For functional impairment at both post-treatment and 6FU, Adapted TSC required significantly fewer sessions to achieve a clinically meaningful improvement compared to Standard TSC. While Adapted TSC required fewer sessions than Standard to achieve a clinically meaningful change, the median number of Adapted TSC sessions to achieve this change ranged from 6 to 9, depending on outcome. Importantly, this dose is higher than that proposed from the adaptation process, which was informed by theory, data, and end-user perspectives (Harvey et al., 2025, 2026). The discrepancy between the number of recommended sessions and those actually delivered by providers reflects the challenges of conducting research in routine practice settings and may indicate that four sessions of Adapted TSC are insufficient when implemented into a real-world setting.

Togther, these findings suggest that the optimal number of sessions for therapists to deliver TSC in routine practice is 8 sessions of Standard TSC and 6 sessions of Adapted TSC to optimize outcomes for the primary outcome of sleep disturbance. However, a greater number of sessions is needed to observe meaningful improvements in psychiatric symptoms and functional impairment compared to sleep outcomes (e.g., 7 to 13 vs. 6 to 8, respectively to optimize outcomes at post-treatment). Similar findings were observed for cumulative session length (see Supplement Table 7), with psychiatric symptoms and functional impairment requiring a greater session length to observe clinically meaningful change compared to sleep outcomes (e.g., 430 to 1078 minutes for sleep outcomes vs. 560 to 1120 minutes for psychiatric symptoms and functional impairment). Furthermore, Standard relative to Adapted TSC required qualitatively greater cumulative session length across outcomes (520 to 1120 minutes for Standard TSC and 430 to 606 minutes for Adapted TSC), but this difference was not significant. The mechanisms through which sleep improvements influence functional impairment and psychiatric symptoms require a higher dose and may take a longer time to observe effects. In other words, while improved sleep outcomes are observed following sleep treatment, the downstream effects on depression, anxiety, and other psychiatric symptoms may take a more sustained period of sleep improvement to become pronounced. These findings are consistent with meta-analyses reporting moderate to large effects of insomnia treatment on improving depression and anxiety symptoms, but these are often assessed at follow-up points after initial sleep improvements have been established (Belleville et al., 2011; Cunningham & Shapiro, 2018; Gebara et al., 2018). Given that evidence-based treatments have variable effect sizes and long-term outcomes (Cuijpers et al., 2023; Hofmann et al., 2012; Van Dis et al., 2020), the present findings give concrete guidelines for the optimal number of sessions to deliver Standard and Adapted TSC.

This study should be interpreted considering several limitations. First, the current study explores the variability in the number of sessions delivered to investigate the optimal session dose. The number of sessions delivered to patients was not systematically manipulated and the actual number of sessions delivered often deviated from the recommended protocol for both intervention arms (e.g., Adapted TSC recommended 4 sessions, but the average was 5; Standard TSC recommended 8 sessions, but the average was 9). However, the goal of the current work was to examine how the number of sessions of treatment affects outcomes, in a real-world treatment setting, as the impact on outcomes reported in clinical trials is not often reported. Second, the aforementioned differences in dropout rate between Adapted and Standard TSC, may have introduced a selection bias, as participants who dropped out may systematically differ from those who remained in the study. Further research is needed to investigate the different reasons for dropout between Standard and Adapted TSC. Third, the results of this study are specific to TSC, and further research is needed to establish whether the findings related to the number of sessions attended and assessment timing reported here generalize to other EBPTs. Finally, as previously mentioned, this study was a secondary analysis that was not preregistered, and therefore, the power calculations were determined from a previously reported trial (Callaway et al., 2023; Sarfan et al., 2023). Given these limitations, findings should be interpreted with caution, with greater emphasis placed on effect sizes and their confidence intervals rather than statistical significance alone.

In summary, the present study provides important insights into an RCT focused on implementing a sleep treatment, TSC, into clinical practice. We found that the effects of TSC were larger for participants who completed the full treatment, although it appeared that a partial dose was also associated with improvements. A substantial number of participants who dropped out showed meaningful improvements by mid-treatment. Although for the parent trial, changes in outcomes were observed across variable assessment timing, effect sizes were larger when completed within 3 months of the target date. Finally, we established a median session dose for Standard and Adapted TSC for clinically meaningful change, providing empirical support for the number of TSC sessions that should be delivered to optimize outcomes.

## Supplementary Material

1

## Figures and Tables

**Table 1:** Patient demographics at pre-treatment and number of sessions by treatment condition (Standard versus Adapted TSC).

Characteristic	Standard TSC (n = 161)		Adapted TSC (*n* = 378)		χ^2^	*p*-value
	n	%	n	%		
Sex					2.71	0.26
Female	94	58.39	238	62.96		
Male	65	40.37	139	36.77		
Missing/declined to answer	2	1.24	1	0.26		
Ethnicity					5.09	0.08
Hispanic or Latino	56	34.78	116	30.69		
Not Hispanic or Latino	101	62.73	260	68.78		
Missing/declined to answer	4	2.48	2	0.53		
Race					8.23	0.31
American Indian/Alaska Native	14	8.7	33	8.73		
Native Hawaiian/Pacific Islander	5	3.11	6	1.59		
Asian	17	10.56	34	8.99		
Black or African American	15	9.32	51	13.49		
White	83	51.55	184	48.68		
More than one race	8	4.97	38	10.05		
Other/category not listed	16	9.94	28	7.41		
Missing/declined to answer	3	1.86	4	1.06		
Education					11.96	0.02
High school graduate or below	47	29.19	85	22.49		
Some or completed college or vocational school	101	62.73	232	61.38		
Some or completed graduate school	9	5.59	57	15.08		
Other/category not listed	1	0.62	1	0.26		
Missing/declined to answer	3	1.86	3	0.79		
	*Mean*	*SD*	*Mean*	*SD*	*t*	*p*-value
Age	42.26	15.14	42.89	15.26	−0.43	0.66
Education (years)	13.62	3.07	14.56	3.57	−3.07	0.002
No. of sessions received (all)^c^	4.55	5.92	3.94	3.59	1.21	0.23
No. of sessions received (completers)^d^	8.01	6.36	5.28	2.92	3.5	0.001

*Note*. Chi-squared was used for categorical variables, and *t* tests were used for continuous variables.

a Some patients endorsed more than one government assistance category.

b Comorbidity was common.

c Number of TSC sessions received by all enrolled patients in the study.

d Number of TSC sessions received by patients who completed treatment.

**Table 2:** Aim 1a: Multilevel modeling results for treatment condition (UC-DT versus TSC) on patient outcomes from pre-to post-treatment by session completion group.

Overall Sample					
Outcome	*b*	SE	p-value	95% CI	*d*
PROMIS-SD	−9.23	0.97	**< 0.001**	−11.14, −7.33	−1.32
PROMIS-SRI	−8.50	1.02	**< 0.001**	−10.49, −6.50	−0.96
SHC	1.15	0.18	**< 0.001**	0.78, 1.51	0.79
DSM-5	−4.19	0.80	**< 0.001**	−5.76, −2.62	−0.52
SDS	−4.87	0.73	**< 0.001**	−6.31, −3.44	−0.66
Completed all sessions					
Outcome	*b*	SE	p-value	95% CI	*d*
PROMIS-SD	−8.98	1.16	**< 0.001**	−11.26, −6.70	−1.25
PROMIS-SRI	−9.01	1.20	**< 0.001**	−11.37, −6.65	−1.11
SHC	1.05	0.21	**< 0.001**	0.63, 1.47	0.74
DSM-5	−4.36	0.92	**< 0.001**	−6.16, −2.56	−0.57
SDS	−5.10	0.86	**< 0.001**	−6.79, −3.42	−0.77
Completed > half of sessions					
Outcome	*b*	SE	p-value	95% CI	*d*
PROMIS-SD	−8.20	3.05	**0.01**	−14.25, −2.14	−0.97
PROMIS-SRI	−8.09	3.00	**0.01**	−14.05, −2.14	−0.74
SHC	1.43	0.57	**0.01**	0.31, 2.56	1.17
DSM-5	−5.97	3.20	0.07	−12.33, 0.39	−0.58
SDS	−4.76	2.11	**0.03**	−8.95, −0.57	−0.58
Completed < half of sessions					
Outcome	*b*	SE	*p*-value	95% CI	*d*
PROMIS-SD	−6.65	3.51	0.06	−13.60, 0.29	−1.04
PROMIS-SRI	−5.96	3.17	0.06	−12.25, 0.33	−0.63
SHC	1.37	0.61	**0.03**	0.16, 2.57	0.81
DSM-5	−5.39	2.39	**0.03**	−10.12, −0.65	−0.33
SDS	−2.56	2.50	0.31	−7.51, 2.39	−0.48

*Note*. Bold indicates significant *p*-values. *b* = time-by-treatment interaction. SE = robust standard errors. PROMIS-SD = PROMIS Sleep Disturbance. PROMIS-SRI = PROMIS Sleep-Related Impairment. SHC = Sleep Health Composite. DSM-5 = DSM-5 Cross-Cutting. SDS = Sheehan Disability Scale. 95% CI = 95% Confidence Interval. Effect sizes are represented with ‘*d*’ and were calculated following Feingold, (2009, equation 5), using unadjusted change scores and raw standard deviations at pre-treatment from each treatment condition.

**Table 3:** Aim 1b: Multilevel modeling results for Adapted vs. Standard TSC on provider perceptions of treatment fit from pre-to post-treatment by session completion group.

Overall Sample					
Outcome	*b*	SE	*p*-value	95% CI	*d*
AIM	−0.10	0.10	0.30	−0.29, 0.09	−0.16
FIM	−0.03	0.13	0.81	−0.29, 0.22	−0.25
IAM	−0.14	0.11	0.20	−0.36, 0.08	−0.08
Completed all sessions					
Outcome	*b*	SE	*p*-value	95% CI	*d*
AIM	−0.08	0.15	0.59	−0.37, 0.21	−0.15
FIM	0.00	0.17	0.97	−0.34, 0.35	0.04
IAM	−0.03	0.16	0.87	−0.34, 0.29	−0.03
Completed > half of sessions					
Outcome	*b*	SE	*p*-value	95% CI	*d*
AIM	−0.39	0.18	0.03	−0.74, −0.03	−0.92
FIM	−0.22	0.23	0.35	−0.68, 0.25	−0.76
IAM	−0.30	0.20	0.13	−0.70, 0.09	−0.49
Completed < half of sessions					
Outcome	*b*	SE	*p*-value	95% CI	*d*
AIM	−0.34	0.20	0.10	−0.73, 0.06	−0.91
FIM	−0.32	0.21	0.13	−0.73, 0.09	−0.87
IAM	−0.37	0.20	0.06	−0.76, 0.02	−0.50

*Note. b* = time-by-treatment interaction. SE = robust standard errors. AIM = Acceptability of Intervention Measure. FIM = Feasibility of Intervention Measure. IAM = Intervention Appropriateness measure. 95% CI = 95% Confidence Interval. *d* = calculated following Feingold (2009, equation 5).

**Table 4:** Aim 3: Multilevel modeling results for treatment condition (UC-DT versus TSC) on patient outcomes from pre-to post-treatment by post.

Outcome	Within 3-months of target					More than 3-months after target				
	*b*	SE	*p*-value	95% CI	*d*	*b*	SE	*p*-value	95% CI	*d*
PROMIS-SD	−10.23	1.46	**<0.001**	−13.12, −7.35	−1.51	−7.43	1.70	**<0.001**	−10.79, −4.07	−0.90
PROMIS-SRI	−9.61	1.36	**<0.001**	−12.30, −6.92	−1.12	−8.77	1.84	**<0.001**	−12.39, −5.15	−1.12
SHC	1.65	0.27	**<0.001**	1.11, 2.19	1.10	0.48	0.33	0.14	−0.17, 1.13	0.25
DSM-5	−5.05	1.08	**<0.001**	−7.18, −2.19	−0.56	−3.84	1.54	**0.01**	−6.87, −0.81	−0.45
SDS	−4.50	1.03	**<0.001**	−6.53, −2.46	−0.74	−4.92	2.39	**<0.001**	−7.70, −2.17	−0.62

*Note*. Bold indicates significant *p*-values. *b* = time-by-treatment interaction. SE = robust standard errors. PROMIS-SD = PROMIS Sleep Disturbance. PROMIS-SRI = PROMIS Sleep-Related Impairment. SHC = Sleep Health Composite. DSM-5 = DSM-5 Cross-Cutting. SDS = Sheehan Disability Scale. 95% CI = 95% Confidence Interval. *d* = calculated following Feingold (2009, equation 5).

**Table 5:** Aim 4: Number of sessions for meaningful clinical change in sleep disturbance, sleep-related impairment, sleep health composite, psychiatric symptoms, and functional impairment by treatment condition (Standard versus Adapted TSC).

Outcome	Standard TSC				Adapted TSC				Hazard Ratio	*p*-value
	Median	95% CI	25%	75%	Median	95% CI	25%	75%		
PROMIS-SD										
Post	8	[6, 9]	5	10	6	[6, 7]	4	9	1.38	0.10
6FU	7	[6, 8]	8	10	6	[5, 6]	4	8	1.44	0.02
PROMIS-SRI										
Post	8	[7, NA^1^]	6	16	7	[6, 8]	5	10	1.57	0.06
6FU	8	[7, 15]	6	15	6	[6, 7]	5	19	1.79	0.004
SHC										
Post	8	[6, NA]	5	16	7	[6, 9]	5	12	1.02	0.95
6FU	8	[6, 10]	5	13	7	[6, 8]	5	9	1.14	0.54
DSM-5										
Post	13	[10, NA]	8	16	9	[8, NA]	5	NA	1.73	0.07
6FU	11	[8, NA]	7	16	9	[7, NA]	5	NA	1.37	0.18
SDS										
Post	11	[8, NA]	8	16	7	[7, 9]	5	11	2.21	0.01
6FU	11	[8, NA]	6	16	7	[6, 8]	5	11	1.78	0.01

1 Some upper bound estimates for the 95% CI and 75% sensitivity analyses were not estimatable due to a smaller sample size for these measures and are represented with NA.

## Data Availability

Data will be made available on request.

## Appendix A. Supplementary data

Supplementary data to this article can be found online at https://doi.org/10.1016/j.brat.2026.104987.
