# Supplementary material for: Patient outcomes and session dose in a randomized controlled trial of the Transdiagnostic Intervention for Sleep and Circadian Dysfunction: Influential factors and methodological considerations
Source: Behav Res Ther. Author manuscript; Available in PMC 2026 May 26. (PMC13209623; doi:10.1016/j.brat.2026.104987)
Supplement: 1 [file NIHMS2178232-supplement-1.docx]

**Supplement File 1**

**Inclusion Criteria**

The inclusion criteria for selecting CMHC sites within these counties to recruit providers and patients were: (1) provision of publicly funded adult mental health outpatient services and (2) support from CMHC leadership. The inclusion criteria for providers were: (1) employed or able to deliver client-facing services to patients within the CMHC; (2) interested in learning and delivering TSC; and (3) volunteered to participate and formally consented to participate.

The inclusion criteria for patients were: (1) aged 18 years and older; (2) met criteria for an SMI per self-report and confirmed by referring provider or administration of the Mini International Neuropsychiatric Interview (MINI; DSM-5, Version 7.0.0) by a licensed clinical social worker on the research team; (3) exhibited a sleep or circadian disturbance as determined by endorsing 4 (quite a bit) or 5 (very much), or the equivalent for reverse scored items, on one or more PROMIS-Sleep Disturbance questions (Buysse et al., 2010; Yu et al., 2012); (4) guaranteed place to sleep for at least two months that was not a shelter; (5) received the standard of care for the SMI and consent to regular communications between the research team and provider; and (6) consented to access their medical record and participate in assessments.

**Exclusion Criteria**

Patients were excluded if they met any of the following criteria: (1) presence of an active and progressive physical illness or neurological degenerative disease directly related to the onset and course of the sleep and circadian problems, or that made participation in the study unfeasible, as assessed by the Checklist of Medical Conditions and Symptoms on the Duke Structured Interview for Sleep Disorders (Carney et al., 2008) and clinical interview; (2) presence of substance abuse/dependence only if it made participation in the study unfeasible; (3) current active intent or plan to commit suicide (those with suicidal ideation were eligible) only if it made participation in the study unfeasible, or homicide risk; (4) night shift work for more than two nights per week in the past three months (i.e., regularly scheduled work from 12 a.m. – 6 a.m.); or (5) pregnant or breastfeeding.

**Aim 4: Optimal Session Length Dosage**

Results of the Kaplan-Meier Survival Analyses, corresponding sensitivity analyses, and hazard ratios from Cox regression models are presented in Supplement Table 4. For clinically meaningful improvement in sleep disturbance at post-treatment, the median cumulative session length was 570 (95% CI [369, 1220]) for Standard TSC, and 490 (95% CI [460, 560]) for Adapted TSC. For clinically meaningful improvement in sleep disturbance at 6-month follow-up, the median cumulative session length was 520 (95% CI [360, 920]) for Standard TSC, and 430 (95% CI [394, 490]) for Adapted TSC. No significant effect of treatment condition was found at post-treatment or 6FU.

For clinically meaningful improvement in sleep-related impairment at post-treatment, the median cumulative session length was 1078 (95% CI [484, NA]) for Standard TSC, and 582 (95% CI [530, 620]) for Adapted TSC. For clinically meaningful improvement in sleep-related impairment at 6-month follow-up, the median cumulative session length was 740 (95% CI [508, NA]) for Standard TSC, and 510 (95% CI [480, 564]) for Adapted TSC. No significant effect of treatment condition was found at post-treatment or 6FU.

For clinically meaningful improvement in the sleep health composite at post-treatment, the median cumulative session length was 600 (95% CI [360, NA]) for Standard TSC, and 560 (95% CI [496, 632]) for Adapted TSC. For clinically meaningful improvement in sleep health composite at 6-month follow-up, the median cumulative session length was 530 (95% CI [484, NA]) for Standard TSC, and 540 (95% CI [476, 610]) for Adapted TSC. No significant effects of treatment condition were found at post-treatment or 6FU.

For clinically meaningful improvement in psychiatric symptoms at post-treatment, the median cumulative session length was 920 (95% CI [520, NA]) for Standard TSC, and 600 (95% CI [560, 800]) for Adapted TSC. For clinically meaningful improvement in psychiatric symptoms at 6-month follow-up, median cumulative session length was 840 (95% CI [520, NA]) for Standard TSC, and 590 (95% CI [540, 728]) for Adapted TSC. No significant effects of treatment condition were found at post-treatment or 6FU.

For clinically meaningful improvement in functional impairment at post-treatment, the median cumulative session length was 1220 (95% CI [508, NA]) for Standard TSC, and 606 (95% CI [554, 728]) for Adapted TSC. For clinically meaningful improvement in functional impairment at 6-month follow-up, the median cumulative session length was 600 (95% CI [520, NA]) for Standard TSC, and 560 (95% CI [490, 632]) for Adapted TSC. No significant effects of treatment condition were found at post-treatment or 6FU.

**Supplement Table 1. Pre-Treatment Patient Demographics by Delayed TSC (UC-DT) compared to Immediate TSC**

| **Characteristic** | **UC-DT (*n* = 263)** | |  | **Immediate (*n* = 276)** | |  |  |
| --- | --- | --- | --- | --- | --- | --- | --- |
|  | ***n*** | ***%*** |  | ***n*** | ***%*** | **χ^2^** | ***p*-value** |
| Sex |  |  |  |  |  | 0.47 | 0.79 |
| Female | 160 | 60.84 |  | 172 | 62.32 |  |  |
| Male | 101 | 38.4 |  | 103 | 37.32 |  |  |
| Missing/declined to answer | 2 | 0.76 |  | 1 | 0.36 |  |  |
| Ethnicity |  |  |  |  |  | 0.78 | 0.68 |
| Hispanic or Latino | 84 | 31.94 |  | 88 | 31.88 |  |  |
| Not Hispanic or Latino | 175 | 66.54 |  | 186 | 67.39 |  |  |
| Missing/declined to answer | 4 | 1.52 |  | 2 | 0.72 |  |  |
| Race |  |  |  |  |  | 5.65 | 0.58 |
| American Indian/Alaska Native | 20 | 7.6 |  | 27 | 9.78 |  |  |
| Native Hawaiian/Pacific Islander | 6 | 2.28 |  | 5 | 1.81 |  |  |
| Asian | 31 | 11.79 |  | 20 | 7.25 |  |  |
| Black or African American | 33 | 12.55 |  | 33 | 11.96 |  |  |
| White | 125 | 47.53 |  | 142 | 51.45 |  |  |
| More than one race | 24 | 9.13 |  | 22 | 7.97 |  |  |
| Other/category not listed | 22 | 8.37 |  | 22 | 7.97 |  |  |
| Missing/declined to answer | 2 | 0.76 |  | 5 | 1.81 |  |  |
| Education |  |  |  |  |  | 2.17 | 0.7 |
| High school graduate or below | 62 | 23.57 |  | 70 | 25.36 |  |  |
| Some or completed college or vocational school | 161 | 61.22 |  | 172 | 62.32 |  |  |
| Some or completed graduate school | 37 | 14.07 |  | 29 | 10.51 |  |  |
| Other/category not listed | 1 | 0.38 |  | 1 | 0.36 |  |  |
| Missing/declined to answer | 2 | 0.76 |  | 4 | 1.45 |  |  |
|  |  |  |  |  |  |  |  |
|  | ***Mean*** | ***SD*** |  | ***Mean*** | ***SD*** | **t** | ***p*-value** |
| Age | 42.26 | 15.14 |  | 42.89 | 15.26 | -0.43 | 0.66 |
| Education (years) | 13.62 | 3.07 |  | 14.56 | 3.57 | -3.07 | 0.002 |
| No. of sessions received (all)^c^ | 4.55 | 5.92 |  | 3.94 | 3.59 | 1.21 | 0.23 |
| No. of sessions received (completers)^d^ | 8.01 | 6.36 |  | 5.28 | 2.92 | 3.5 | 0.001 |
| *Note.* Chi-squared was used for categorical variables, and *t* tests were used for continuous variables. ^a^Some patients endorsed more than one government assistance category. ^b^Comorbidity was common. ^c^Number of TSC sessions received by all enrolled patients in the study. ^d^Number of TSC sessions received by patients who completed treatment. | | | | | | | |

**Supplement Table 2. Provider Demographics by TSC Treatment Condition (Standard versus Adapted TSC) at Pre-Treatment**

| **Characteristic** | **Standard TSC (*n* = 53)** | |  | **Adapted TSC (*n* = 124)** | |  | |
| --- | --- | --- | --- | --- | --- | --- | --- |
|  | ***n*** | ***%*** |  | ***n*** | ***%*** | **χ^2^** | ***p*-value** |
| Sex |  |  |  |  |  | 2.66 | 0.45 |
| Female | 42 | 79.25 |  | 91 | 73.39 |  |  |
| Male | 5 | 9.43 |  | 8 | 6.45 |  |  |
| Other/category not listed | 0 | 0 |  | 2 | 1.61 |  |  |
| Missing/declined to answer | 6 | 11.32 |  | 23 | 18.55 |  |  |
| Ethnicity |  |  |  |  |  | 1.21 | 0.55 |
| Hispanic or Latino | 16 | 30.19 |  | 28 | 22.58 |  |  |
| Not Hispanic or Latino | 25 | 47.17 |  | 67 | 54.03 |  |  |
| Missing/declined to answer | 12 | 22.64 |  | 29 | 23.39 |  |  |
| Race |  |  |  |  |  | 1.59 | 0.95 |
| American Indian/Alaska Native | 1 | 1.89 |  | 2 | 1.61 |  |  |
| Native Hawaiian or Pacific Islander | 0 | 0 |  | 1 | 0.81 |  |  |
| Asian | 6 | 11.32 |  | 16 | 12.9 |  |  |
| Black or African American | 4 | 7.55 |  | 5 | 4.03 |  |  |
| White | 28 | 52.83 |  | 65 | 52.42 |  |  |
| More than one race | 3 | 5.66 |  | 6 | 4.84 |  |  |
| Missing/declined to answer | 11 | 20.75 |  | 29 | 23.39 |  |  |
| Degree Type^a^ |  |  |  |  |  | 15.04 | 0.04 |
| Marriage and Family Therapy | 18 | 33.96 |  | 20 | 16.13 |  |  |
| Psychology | 4 | 7.55 |  | 14 | 11.29 |  |  |
| Social Work | 16 | 30.19 |  | 40 | 32.26 |  |  |
| Nursing | 0 | 0 |  | 9 | 7.26 |  |  |
| Medical | 2 | 3.77 |  | 1 | 0.81 |  |  |
| Occupational Therapy | 0 | 0 |  | 6 | 4.84 |  |  |
| Other/category not listed | 5 | 9.43 |  | 10 | 8.06 |  |  |
| Missing | 8 | 15.09 |  | 25 | 20.16 |  |  |
| Therapeutic Approach^a^ |  |  |  |  |  | 2.79 | 0.9 |
| Client Centered | 32 | 60.38 |  | 79 | 63.71 |  |  |
| Family Systems | 10 | 18.87 |  | 27 | 21.77 |  |  |
| CBT | 33 | 62.26 |  | 59 | 47.58 |  |  |
| Psychodynamic | 11 | 20.75 |  | 27 | 21.77 |  |  |
| Humanistic | 3 | 5.66 |  | 8 | 6.45 |  |  |
| Integrative/Holistic | 1 | 1.89 |  | 5 | 4.03 |  |  |
| None | 1 | 1.89 |  | 3 | 2.42 |  |  |
| Missing/declined to answer | 9 | 16.98 |  | 27 | 21.77 |  |  |
| Licensure |  |  |  |  |  | 1.52 | 0.47 |
| Licensed | 27 | 50.94 |  | 65 | 52.42 |  |  |
| Not Licensed | 19 | 35.85 |  | 35 | 28.23 |  |  |
| Missing/declined to answer | 7 | 13.21 |  | 24 | 19.35 |  |  |
|  |  |  |  |  |  |  |  |
|  | ***Mean*** | ***SD*** |  | ***Mean*** | ***SD*** | **t** | ***p*-value** |
| Age | 40.19 | 10.48 |  | 41.03 | 10.78 | 0.98 | 0.33 |
| Caseload | 40.56 | 26.6 |  | 30 | 32.66 | -1 | 0.32 |
| Employment Duration | 3.25 | 3.02 |  | 4.66 | 5.24 | -2.04 | 0.04 |
| Years Since Degree Earned | 9.49 | 7.93 |  | 9.86 | 8.5 | -0.25 | 0.81 |
| ***Note****.* ^a^Some providers endorsed more than one degree type and therapeutic approach. Chi-squared was used for categorical variables, and *t* tests were used for continuous variables. CBT = cognitive behavioral therapy. Caseload = number of clients on caseload. Employment duration = length of time employed at current CMHC in years. | | | | | | | |

| **Standard** | **Adapted** |
| --- | --- |
| Core modules | |
| *Core Module 1a*: Targets irregular sleep-wake times | *Core Module 1*: Targets irregular sleep-wake times |
| *Core Module 1b*: Targets difficulty winding down | *Core Module 2*: Targets difficulty winding down |
| *Core Module 1c:* Targets difficulty waking up | *Core Module 3*: Targets difficulty waking up |
| *Core Module 2*: Aims to reduce daytime impairment | *Core Module 4*: Aims to reduce daytime impairment |
| *Core Module 3*: Focuses on unhelpful beliefs about sleep | *Core Module 5*: Promotes maintenance of change |
| *Core Module 4:*  Promotes maintenance of change | -- |
| Optional modules | |
| *Optional Module 1*: Addresses poor sleep efficiency via stimulus control and sleep restriction | *Optional Module 1*: Focuses on reducing sleep-related worry |
| *Optional Module 2*: Helps patients reduce time in bed | -- |
| *Optional Module 3*: Addresses delayed or advanced phase problems (e.g., going to sleep later than desired or waking up earlier than desired) | -- |
| *Optional Module 4*: Focuses on reducing sleep-related worry | -- |
| *Optional Module 5*: Promotes compliance with Continuous Positive Airways Pressure (CPAP) for patients with sleep apnea | -- |
| *Optional Module 6:* Helps patients negotiate sleep in complicated environments (e.g., noise from bed/roommates, traffic noise, streetlight entering the bedroom) | -- |
| *Optional Module 7*: Helps patients who experience nightmares | -- |

**Supplement Table 3. Descriptions of Cross-cutting, Core, and Optional Modules Offered for the Standard and Adapted Conditions**

**Cross-cutting modules**: Case formulation, Sleep and circadian education, Motivational enhancement, Goal setting

**Supplement Table 4. Means, Standard Deviations, and Effect Sizes for Primary and Secondary Patient Outcomes by Session Completion Group**

|  | **Overall Sample** | | | | | | | |  |  |
| --- | --- | --- | --- | --- | --- | --- | --- | --- | --- | --- |
|  | **Pre-Treatment** | | | | **Post-Treatment** | | | |  |  |
|  | **UC-DT (N = 263)** | | **TSC (N = 276)** | | **UC-DT** | | **TSC** | | ***d*** |  |
|  | **Mean** | **SD** | **Mean** | **SD** | **Mean** | **SD** | **Mean** | **SD** |  |  |
| PROMIS-SD* | 62.62 | 7.71 | 62.74 | 7.28 | 61.51 | 8.38 | 52.09 | 10.52 | -1.32 |  |
| PROMIS-SRI | 61.51 | 8.28 | 61.64 | 8.82 | 60.37 | 8.51 | 52.00 | 10.86 | -0.96 |  |
| SHC | 2.15 | 1.37 | 2.08 | 1.42 | 2.34 | 1.49 | 3.40 | 1.59 | 0.79 |  |
| DSM-5 | 23.81 | 9.37 | 24.12 | 8.88 | 22.69 | 8.79 | 18.46 | 10.30 | -0.52 |  |
| SDS | 12.83 | 6.90 | 12.67 | 7.44 | 12.03 | 7.25 | 6.93 | 6.37 | -0.66 |  |
|  | **Completed all sessions** | | | | | | | |  |  |
|  | **Pre-Treatment** | | | | **Post-Treatment** | | | |  |  |
|  | **UC-DT (N = 127)** | | **TSC (N = 144)** | | **UC-DT** | | **TSC** | | ***d*** |  |
|  | **Mean** | **SD** | **Mean** | **SD** | **Mean** | **SD** | **Mean** | **SD** |  |  |
| PROMIS-SD* | 62.47 | 7.70 | 61.82 | 7.23 | 61.47 | 7.86 | 51.84 | 10.39 | -1.25 |  |
| PROMIS-SRI | 61.57 | 7.94 | 61.24 | 8.30 | 60.66 | 8.35 | 51.09 | 11.10 | -1.11 |  |
| SHC | 2.02 | 1.27 | 2.12 | 1.34 | 2.24 | 1.30 | 3.35 | 1.57 | 0.74 |  |
| DSM-5 | 22.83 | 9.64 | 23.23 | 8.63 | 22.77 | 8.27 | 17.36 | 9.47 | -0.57 |  |
| SDS | 12.76 | 6.73 | 12.65 | 6.80 | 11.50 | 6.80 | 6.14 | 6.24 | -0.77 |  |
|  | **Completed > half of sessions** | | | | | | | |  |  |
|  | **Pre-Treatment** | | | | **Post-Treatment** | | | | |  |
|  | **UC-DT (N = 26)** | | **TSC (N = 37)** | | **UC-DT** | | **TSC** | | ***d*** |  |
|  | **Mean** | **SD** | **Mean** | **SD** | **Mean** | **SD** | **Mean** | **SD** |  |  |
| PROMIS-SD* | 64.08 | 6.66 | 62.76 | 7.18 | 60.48 | 8.35 | 51.94 | 10.15 | -0.97 |  |
| PROMIS-SRI | 63.11 | 7.47 | 63.71 | 9.56 | 62.20 | 8.31 | 55.45 | 10.67 | -0.74 |  |
| SHC | 2.29 | 1.40 | 2.03 | 1.34 | 2.55 | 1.74 | 3.84 | 1.77 | 1.17 |  |
| DSM-5 | 24.08 | 9.68 | 26.00 | 9.65 | 25.67 | 9.75 | 22.00 | 11.86 | -0.58 |  |
| SDS | 12.92 | 6.11 | 14.16 | 7.89 | 12.54 | 7.67 | 9.10 | 6.28 | -0.58 |  |
| **Completed ≤ half of sessions** | | | | | | | | | |  |
|  | **Pre-Treatment** | | | | **Post-Treatment** | | | | |  |
|  | **UC-DT (N = 34)** | | **TSC (N = 51)** | | **UC-DT** | | **TSC** | | ***d*** |  |
|  | **Mean** | **SD** | **Mean** | **SD** | **Mean** | **SD** | **Mean** | **SD** |  |  |
| PROMIS-SD* | 62.46 | 7.53 | 64.25 | 7.13 | 61.52 | 8.72 | 55.95 | 12.59 | -1.04 |  |
| PROMIS-SRI | 60.93 | 8.32 | 62.30 | 9.30 | 59.47 | 9.57 | 54.83 | 7.90 | -0.63 |  |
| SHC | 2.26 | 1.40 | 1.72 | 1.53 | 2.23 | 1.55 | 2.92 | 1.51 | 0.81 |  |
| DSM-5 | 23.21 | 9.66 | 26.04 | 9.31 | 21.36 | 8.72 | 21.15 | 10.85 | -0.33 |  |
| SDS | 12.06 | 6.78 | 12.94 | 8.56 | 13.06 | 7.59 | 10.08 | 6.12 | -0.48 |  |
| *Note.* *indicates primary outcome. PROMIS-SD = PROMIS Sleep Disturbance. PROMIS-SRI = PROMIS Sleep-Related Impairment. SHC = Sleep Health Composite (note, scored such that higher scores indicate better sleep health). DSM-5 = DSM-5 Cross-Cutting. SDS = Sheehan Disability Scale. TSC = Transdiagnostic Intervention for Sleep and Circadian Dysfunction. UC-DT = usual care followed by delayed treatment with TSC. Effect sizes are represented with ‘*d*’ and were calculated following Feingold (2009, equation 5), using unadjusted change scores and raw standard deviations at pre-treatment from each treatment condition. | | | | | | | | | |  |

**Supplement Table 5. Means, Standard Deviations, and Effect Sizes for Primary and Secondary Provider Outcomes by Session Completion Group**

|  | **Overall Sample** | | | | | | | |  |
| --- | --- | --- | --- | --- | --- | --- | --- | --- | --- |
|  | **Pre-Treatment** | | | | **Post-Treatment** | | | |  |
|  | **Standard (N = 161)** | | **Adapted (N = 378)** | | **Standard TSC** | | **Adapted TSC** | | ***d*** |
|  | **Mean** | **SD** | **Mean** | **SD** | **Mean** | **SD** | **Mean** | **SD** |  |
| AIM* | 4.72 | 0.39 | 4.67 | 0.57 | 4.76 | 0.51 | 4.64 | 0.53 | -0.16 |
| IAM | 4.69 | 0.47 | 4.69 | 0.56 | 4.68 | 0.57 | 4.54 | 0.58 | -0.25 |
| FIM | 4.62 | 0.46 | 4.56 | 0.62 | 4.67 | 0.65 | 4.58 | 0.55 | -0.08 |
|  | **Completed all sessions** | | | | | | | |  |
|  | **Pre-Treatment** | | | | **Post-Treatment** | | | |  |
|  | **Standard (N = 32)** | | **Adapted (N = 239)** | | **Standard TSC** | | **Adapted TSC** | | ***d*** |
|  | **Mean** | **SD** | **Mean** | **SD** | **Mean** | **SD** | **Mean** | **SD** |  |
| AIM* | 4.70 | 0.44 | 4.68 | 0.59 | 4.75 | 0.62 | 4.66 | 0.49 | -0.15 |
| IAM | 4.79 | 0.43 | 4.70 | 0.58 | 4.66 | 0.63 | 4.55 | 0.57 | 0.04 |
| FIM | 4.66 | 0.48 | 4.58 | 0.64 | 4.68 | 0.69 | 4.59 | 0.50 | -0.03 |
|  | **Completed > half of sessions** | | | | | | | |  |
|  | **Pre-Treatment** | | | | **Post-Treatment** | | | | |
|  | **Standard (N = 36)** | | **Adapted (N = 27)** | | **Standard TSC** | | **Adapted TSC** | | ***d*** |
|  | **Mean** | **SD** | **Mean** | **SD** | **Mean** | **SD** | **Mean** | **SD** |  |
| AIM* | 4.61 | 0.49 | 4.78 | 0.41 | 4.76 | 0.42 | 4.53 | 0.59 | -0.92 |
| IAM | 4.53 | 0.55 | 4.78 | 0.41 | 4.68 | 0.50 | 4.58 | 0.56 | -0.76 |
| FIM | 4.52 | 0.51 | 4.78 | 0.35 | 4.64 | 0.66 | 0.14 | 4.69 | -0.49 |
| **Completed ≤ half of sessions** | | | | | | | | | |
|  | **Pre-Treatment** | | | | **Post-Treatment** | | | | |
|  | **Standard (N = 45)** | | **Adapted (N = 50)** | | **Standard TSC** | | **Adapted TSC** | | ***d*** |
|  | **Mean** | **SD** | **Mean** | **SD** | **Mean** | **SD** | **Mean** | **SD** |  |
| AIM* | 4.68 | 0.41 | 4.81 | 0.35 | 4.68 | 0.51 | 4.49 | 0.78 | -0.91 |
| IAM | 4.70 | 0.49 | 4.79 | 0.40 | 4.61 | 0.66 | 4.37 | 0.69 | -0.87 |
| FIM | 4.66 | 0.45 | 4.60 | 0.50 | 4.64 | 0.66 | 4.33 | 0.79 | -0.50 |
| *Note.* *indicates primary outcome. AIM = Acceptability of Intervention Measure. FIM = Feasibility of Intervention Measure. IAM = Intervention Appropriateness measure. TSC = Transdiagnostic Intervention for Sleep and Circadian Dysfunction. UC-DT = usual care followed by delayed treatment with TSC. Effect sizes are represented with ‘*d*’ and were calculated following Feingold (2009, equation 5), using unadjusted change scores and raw standard deviations at pre-treatment from each treatment condition. | | | | | | | | | |

**Supplement Table 6. Means, Standard Deviations, and Effect Sizes for Primary and Secondary Patient Outcomes by Post-assessment Timing (Within 3 Months of Target versus Greater than 3 Months of Target)**

|  | **Pre-Treatment** | | | | **Post-Treatment** | | | |  |
| --- | --- | --- | --- | --- | --- | --- | --- | --- | --- |
| **Within 3 months** |  | |  | |  | |  | |  |
|  | **UC-DT (n = 62)** | | **TSC (n = 82)** | | **UC-DT (n = 62)** | | **TSC (n = 82)** | | ***d*** |
|  | **Mean** | **SD** | **Mean** | **SD** | **Mean** | **SD** | **Mean** | **SD** |  |
| PROMIS-SD* | 62.09 | 7.87 | 61.80 | 6.90 | 60.63 | 7.82 | 50.11 | 10.11 | -1.51 |
| PROMIS-SRI | 60.71 | 8.17 | 60.59 | 8.60 | 60.42 | 8.79 | 50.62 | 10.75 | -1.12 |
| SHC | 2.21 | 1.33 | 2.10 | 1.46 | 2.23 | 1.34 | 3.73 | 1.63 | 1.10 |
| DSM-5 | 23.29 | 9.51 | 22.73 | 9.05 | 22.68 | 9.18 | 17.07 | 10.06 | -0.56 |
| SDS | 12.47 | 7.26 | 11.79 | 6.28 | 11.26 | 7.10 | 6.09 | 6.13 | -0.74 |
|  |  |  |  |  |  |  |  |  |  |
| **More than 3 months** |  |  |  |  |  |  |  |  |  |
|  | **UC-DT (n = 57)** | | **TSC (n = 60)** | | **UC-DT (n = 57)** | | **TSC (n = 60)** | | ***d*** |
|  | **Mean** | **SD** | **Mean** | **SD** | **Mean** | **SD** | **Mean** | **SD** |  |
| PROMIS-SD* | 61.76 | 7.11 | 62.54 | 8.03 | 60.94 | 8.42 | 54.37 | 10.69 | -0.90 |
| PROMIS-SRI | 61.08 | 7.71 | 63.62 | 7.82 | 59.85 | 8.52 | 53.64 | 11.24 | -1.12 |
| SHC | 2.09 | 1.24 | 2.23 | 1.39 | 2.50 | 1.45 | 3.04 | 1.44 | 0.25 |
| DSM-5 | 23.37 | 9.65 | 24.16 | 9.08 | 22.41 | 7.86 | 20.07 | 9.54 | -0.45 |
| SDS | 11.32 | 5.71 | 13.35 | 7.28 | 10.27 | 6.61 | 7.52 | 6.52 | -0.62 |
| *Note.* *indicates primary outcome. PROMIS-SD = PROMIS Sleep Disruption. PROMIS-SD = PROMIS Sleep Disturbance. PROMIS-SRI = PROMIS Sleep-Related Impairment. SHC = Sleep Health Composite. DSM-5 = DSM-5 Cross-Cutting. SDS = Sheehan Disability Scale. TSC = Transdiagnostic Intervention for Sleep and Circadian Dysfunction. UC-DT = usual care followed by delayed treatment with TSC. Effect sizes are represented with ‘*d*’ and were calculated following Feingold (2009, equation 5), using unadjusted change scores and raw standard deviations at pre-treatment from each treatment condition. | | | | | | | | | |

| \| **Supplement Table 7. Aim 4: Optimal Cumulative Session Length for Meaningful Clinical Change in Sleep Disturbance, Sleep-related Impairment, Sleep Health Composite, Psychiatric Symptoms, and Functional Impairment by Treatment Condition (Standard versus Adapted TSC)** \| \| \| \| \| \| \| \| \| \| \| \| \| \| --- \| --- \| --- \| --- \| --- \| --- \| --- \| --- \| --- \| --- \| --- \| --- \| --- \| \|  \| **Standard TSC** \| \| \| \|  \| **Adapted TSC** \| \| \| \|  \|  \|  \| \| **Outcome** \| **Median** \| **95% CI** \| **25%** \| **75%** \|  \| **Median** \| **95% CI** \| **25%** \| **75%** \|  \| **Hazard Ratio** \| ***p*-value** \| \| PROMIS-SD \|  \|  \|  \|  \|  \|  \|  \|  \|  \|  \|  \|  \| \| Post \| 570 \| [369, 1220] \| 300 \| 1078 \|  \| 490 \| [460, 560] \| 350 \| 650 \|  \| 1.34 \| 0.27 \| \| 6FU \| 520 \| [360, 920] \| 300 \| 920 \|  \| 430 \| [394, 490] \| 290 \| 606 \|  \| 1.31 \| 0.22 \| \| PROMIS-SRI \|  \|  \|  \|  \|  \|  \|  \|  \|  \|  \|  \|  \| \| Post \| 1078 \| [484, NA] \| 360 \| 1254 \|  \| 582 \| [530, 620] \| 390 \| 920 \|  \| 1.30 \| 0.41 \| \| 6FU \| 740 \| [508, NA] \| 360 \| 1220 \|  \| 510 \| [480, 564] \| 360 \| 650 \|  \| 1.60 \| 0.08 \| \| SHC \|  \|  \|  \|  \|  \|  \|  \|  \|  \|  \|  \|  \| \| Post \| 600 \| [360, NA] \| 350 \| 1254 \|  \| 560 \| [496, 632] \| 370 \| 736 \|  \| 1.28 \| 0.45 \| \| 6FU \| 530 \| [484, NA] \| 350 \| 1254 \|  \| 540 \| [476, 610] \| 360 \| 720 \|  \| 1.12 \| 0.70 \| \| DSM-5 \|  \|  \|  \|  \|  \|  \|  \|  \|  \|  \|  \|  \| \| Post \| 920 \| [520, NA] \| 508 \| 1254 \|  \| 600 \| [560, 800] \| 490 \| 1458 \|  \| 1.21 \| 0.59 \| \| 6FU \| 840 \| [520, NA] \| 484 \| 1254 \|  \| 590 \| [540, 728] \| 394 \| NA \|  \| 1.39 \| 0.29 \| \| SDS \|  \|  \|  \|  \|  \|  \|  \|  \|  \|  \|  \|  \| \| Post \| 1220 \| [508, NA] \| 508 \| 1320 \|  \| 606 \| [554, 728] \| 420 \| 800 \|  \| 1.61 \| 0.20 \| \| 6FU \| 600 \| [520, NA] \| 500 \| 1078 \|  \| 560 \| [490, 632] \| 360 \| 736 \|  \| 1.26 \| 0.38 \| |
| --- | --- | --- | --- | --- | --- | --- | --- | --- | --- | --- | --- | --- | --- | --- | --- | --- | --- | --- | --- | --- | --- | --- | --- | --- | --- | --- | --- | --- | --- | --- | --- | --- | --- | --- | --- | --- | --- | --- | --- | --- | --- | --- | --- | --- | --- | --- | --- | --- | --- | --- | --- | --- | --- | --- | --- | --- | --- | --- | --- | --- | --- | --- | --- | --- | --- | --- | --- | --- | --- | --- | --- | --- | --- | --- | --- | --- | --- | --- | --- | --- | --- | --- | --- | --- | --- | --- | --- | --- | --- | --- | --- | --- | --- | --- | --- | --- | --- | --- | --- | --- | --- | --- | --- | --- | --- | --- | --- | --- | --- | --- | --- | --- | --- | --- | --- | --- | --- | --- | --- | --- | --- | --- | --- | --- | --- | --- | --- | --- | --- | --- | --- | --- | --- | --- | --- | --- | --- | --- | --- | --- | --- | --- | --- | --- | --- | --- | --- | --- | --- | --- | --- | --- | --- | --- | --- | --- | --- | --- | --- | --- | --- | --- | --- | --- | --- | --- | --- | --- | --- | --- | --- | --- | --- | --- | --- | --- | --- | --- | --- | --- | --- | --- | --- | --- | --- | --- | --- | --- | --- | --- | --- | --- | --- | --- | --- | --- | --- | --- | --- | --- | --- | --- | --- | --- | --- | --- | --- | --- | --- | --- | --- | --- | --- | --- | --- | --- | --- | --- | --- | --- | --- | --- | --- | --- | --- | --- | --- | --- | --- | --- | --- | --- | --- | --- |
